# Multifunctional Characteristics of Carbon Fibers Modified with Imidazolium Ionic Liquids

**DOI:** 10.3390/molecules27207001

**Published:** 2022-10-18

**Authors:** Bilal Ghafoor, Henri Stephan Schrekker, Sandro Campos Amico

**Affiliations:** 1 PPGE3M, Federal University of Rio Grande do Sul, Porto Alegre 91501-970, RS, Brazil; 2 Laboratory of Technological Processes and Catalysis, Institute of Chemistry, Federal University of Rio Grande do Sul, Porto Alegre 91501-970, RS, Brazil

**Keywords:** task-specific ionic liquid, carbon fiber recycling, electrical resistivity, interfacial bonding, epoxy

## Abstract

A multifunctional designing approach is of great importance for advanced composite applications. This study assessed the use of ionic liquids (ILs) to modify the surface of carbon fiber (CF) and impart multifunctional characteristics to it. For that, ethanolic solutions of different ILs, 1-butyl-3-methylimidazolium bis(trifluoromethylsulfonyl)imide, 1-butyl-3-methylimidazolium chloride and 1-(2-hydroxyethyl)-3-methylimidazolium chloride, at different concentrations, were used to treat the CF. Fourier-transform infrared spectroscopy confirmed the presence of IL on the CF surface. The contact angle for 1% *w*/*v* IL-treated CF and DGEBA epoxy decreased by up to 35%, corresponding to an increase in surface energy of fiber, accompanied by an increase of 91% in interfacial shear strength. These enhancements were achieved with the hydroxy-functionalized IL, showing the tunability of CF properties through the *N*-imidazolium substituent. An increase in crystallite size along the basal plane was also found due to the ordering of the graphitic structure on the surface. Moreover, there was a decrease in electrical resistivity of 77%. In all, the imidazolium ILs were considered a promising approach to induce multifunctional characteristics, namely enhanced interfacial strength and electrical conductivity, to unsized CF, which can also be beneficial for recycled fibers without deteriorating their inherent surface properties.

## 1. Introduction

A considerable effort to increase the use of more sustainable materials in various fields is of prime interest. The neoteric sense of ionic liquids (ILs) can be understood by multiple aspects like low flammability, neglectable volatility and high thermal stability, providing safe and robust alternatives to traditional organic solvents. The importance of ILs in terms of design flexibility is impressive, including structural changes in the IL’s cation, anion, alkyl chain and functional group. Factors like size and asymmetry of their ions contribute to complex interactions, including dipole–dipole, dipole-induced dipole, dispersion and hydrogen bonding. The structural and chemical characteristics of ILs and their interactions with the liquid–solid interface are a result of these complex Coulombic and intermolecular interactions [1].

Imidazolium ILs, in particular, have good transport properties and high charge carrier capacities and ionic mobilities [2]. Their surface activity is mainly dependent on the molecular structure, i.e., cation, anion, *N*-alkyl chain length and functional group. Their interactions are predominantly of ion-ion (cation-anion) and hydrogen bonding nature (anion-imidazolium cation C_2_-hydrogen) [3]. Coulomb forces are dominant, and the imidazolium cation provides multiple sites for the anion interaction, which is possible from above and below the imidazolium ring as the preferable and thermodynamically stable site. The imidazolium ring C_2_-hydrogen has an acidic character that favors hydrogen bonding with an anion through in-plane conformation [4].

Currently, the ILs have been mostly associated with electrochemical, chemical and, to some extent, optical applications [5], and not much with structural composite materials. Carbon fiber reinforced polymer composites (CFRP) are being used in an ever-growing number of applications in aerospace, automotive, energy and other sectors due to excellent specific strength and functional characteristics. As a side-effect, an ever larger amount of CFRP waste is being discarded at the end of the component life [6].

Different recycling methods for CFRP are being developed to mitigate the impact, including thermal (e.g., pyrolysis, fluidized bed recycling), chemical (e.g., solvolysis, hydrolysis) and electrochemical routes [7,8,9,10,11,12]. Indeed, recycling is a difficult process for composites prepared with thermoset polymer matrices, and the resulting recovered fibers commonly have short lengths and hence low aspect ratios. Nevertheless, many sectors have plans for the use of recycled carbon fibers (rCF) as in urban air mobility, utility poles to trench covers, engine cradles [13], fuel cells [14], wind turbine blades [15], and cementitious mortar to increase mechanical properties [16].

The recycling process also modifies the surface characteristics of carbon fibers since it removes the sizing used to increase compatibility and interfacial adhesion between rCF and the polymer matrix. This has a direct impact on the mechanical properties of the future recycled CFRP [17]. In some cases, rCF without any further surface modification is applied for producing composites [18,19], whereas some studies focused on the treatment of rCF with nitric acid and a coupling agent [20,21,22,23], plasma [17], superheated steam [14], steam/air [24], and polymer sizing [25], which are expected to partly recover the reinforcing potential of the original fiber.

The microcrystalline structure of CF consists of layers of sp^2^ hybridized carbon atoms arranged in a regular hexagonal pattern similar to graphite structure arranged parallel to each other in a regular pattern having atom in plane covalently bonded atoms and van der Waals forces in the transverse direction of the plane. Highly delocalized π-electrons are evident from graphene layers aligned parallel to the fiber axis, and they have a strong influence on the surface properties of carbon fiber. The overall morphology of rCF remains the same, and the changes are observed as increased surface defects, reduced lateral crystallite size, and decreased surface oxygen concentration that might decrease the interfacial shear strength (IFSS) with a polymer [26]. rCF from supercritical methanol has a reduced tensile strength of 9% and an IFSS of 20% due to the removal of some functional groups [27], rCF from an electrochemical method retained 80% of the tensile strength with no change of oxygenated groups on the fiber surface but with a loss in crystallinity [28], and rCF from pyrolysis showed poorer mechanical properties due to the presence of residue on the surface, although the surface morphology remained the same [29].

**Figure 1:**
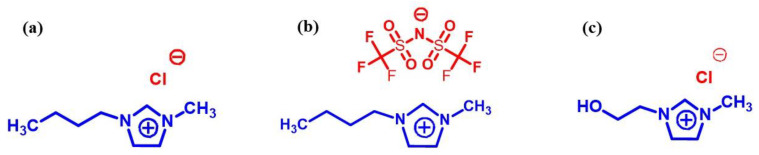
Chemical structures of IL studied: (**a**) C_4_MImCl, (**b**) C_4_MImNTf_2_, (**c**) C_2_OHMImCl.

In a previous study of our group, 10% *w*/*v* of IL was used to treat CF, focusing on the effect of hydrophilic and hydrophobic anions [30]. The successful non-covalent modification of unsized carbon fiber in a mild treatment with ILs has opened new alternatives for the CF treatment without harming its inherent properties. An increase in surface free energy, a decrease in contact angle and an enhanced compatibility with the epoxy matrix in terms of interfacial shear strength were observed. Other reports on the functionalization of CF with IL include the use of 10 wt% of a single IL, 1-butyl-methylimidazolium chloride, as a sizing agent for CF, in which an improved CF/epoxy adhesion strength has been reported [31]. Another study assessed the CF-functionalization with concentrated 1-ethyl-3-methylimidazolium bis(trifluoromethylsulfonyl)imide under microwave irradiation at 50 °C and reported an improvement in CF/epoxy interfacial shear strength [32]. The current study follows on that research, addressing issues like the optimization of the IL concentration (from 0.25% to 3% *w*/*v*) and the effect of different functional groups of the *N*-alkyl side chain of the cation on the surface interactions on the microcrystalline structure of CF, and also assessing the treatment effect on electrical resistivity, focusing on multifunctional characteristics (Figure 1).

## 2. Experiment

### 2.1. Materials

Commercially available carbon fiber roving (modulus = 280 GPa; tensile strength = 4.8 GPa; density = 1.78 g/cm^3^; filament diameter = 6.6 µm) (SIGRAFIL C T50-4.8/280-UN) without sizing was selected as the base material to emulate a rCF. 1-butyl-3-methylimidazolium bis(trifluoromethanesulfonyl)imide (C_4_MImNTf_2_; purity > 99%; water content of 500 ppm; liquid) and 1-butyl-3-methyl imidazolium chloride (C_4_MImCl; purity > 98%; water content < 1%; solid) were acquired from Sigma-Aldrich. The 1-(2-hydroxyethyl)-3-methylimidazolium chloride (C_2_OHMImCl; purity > 98%; solid) was synthesized, and its ^1^H NMR spectrum (shown as Figure S3 in Supplementary Materials), and characterization data were in agreement with those reported [33] Anhydrous ethanol (99.5% purity) was purchased from Sigma-Aldrich. DGEBA epoxy resin (AR260) and hardener (AH260) purchased from e-composites (Brazil) were used for contact angle measurements and to produce samples for pull-out testing.

### 2.2. Fiber Treatment Process

As-received CF (designated as CF) roving was cut (12 cm length), placed in a beaker containing 50 mL of ethanol for 10 min, followed by manual stirring for 4–5 min. The treated CF roving was squeeze dried followed by heating in an oven with air circulation for 30–45 min at 60 °C to remove excess solvent. CF were then treated by immersion in an ethanolic IL solution (50 mL), at different concentrations, for 10 min at room temperature. Afterwards, the samples were removed and dried in a vacuum oven for 30 min at 60 °C. The CF treated with C_4_MImCl, C_4_MImNTf_2_ and C_2_OHMImCl were designated as CF-Cl, CF-NTf_2_ and CF-OH, respectively. CF was washed with ethanol (designated as CF-W) to measure its interfacial strength and to establish differences due to the ethanol treatment on CF. This is the same procedure followed in our previous study [30].

### 2.3. Characterization Techniques

Fourier-transform infrared spectroscopy was performed with Nicolet 6700 equipment in the 750–3500 cm^−1^ range with 120 scans at a constant spectral resolution of 4 cm^−1^ in ATR mode using a germanium crystal. Scanning electron microscopy was carried out in a Carl Zeiss EVO MA10 electron microscope operating at 10 kV, with a tungsten filament current of 2.004 A, a probe current of 20 pA and a working distance of 5.5 mm. Samples were gold-coated, and images were acquired in secondary electron imaging mode. The characteristic X-ray detector (EDS) was used for elemental composition analysis and compositional mapping of the CF samples. The thermal stability of the samples (minimum 10 mg each) was determined using TA instruments (TGA Q50) in the 30 °C to 700 °C range, at a heating rate of 10 °C/min under a nitrogen atmosphere.

The electrical properties of single carbon fibers were measured by the transfer length method (TLM) according to the procedure described in [34]. A single fiber was placed onto a laboratory glass slide, and electrical contacts were made with small droplets of colloidal silver paste (60 ± 1% Ag, sheet resistance: 0.02–0.05 Ω mm^2^ (25 µm)). The two-terminal resistance (R) between the contacts separated by an increasing distance was measured with a multimeter (MD-6200). Then, R was plotted against the distance between the electrical contacts and, by linear fitting, the contact resistance (*R_c_*) was calculated from the y-intercept and the electrical resistivity (*ρ_el_*) from the slope, based on Equation (1). Two samples of each CF were tested, and an average value was reported.

(1) $$R=\left(\rho_{el}A^{-1}\right)\cdot L+R_{c}$$

where *A* is the CF cross-section (equal to 38.5 μm^2^).

Carbon fiber X-ray diffraction analysis was performed by first grinding it to powder form with a mortar. The grounded fiber was transferred to a glass substrate, ensuring the initial zero angle for analysis in a Rigaku equipment, model Ultima V (Cu K_α_ radiation with 0.1541 nm). XRD signals were collected in a 2θ range from 10° to 60° with a step size of 0.05° in a continuous scanning mode operating at 40 kV and 17 mA. The Scherrer equation (Equation (2)) was used to estimate the crystallite size.

(2) $$L_{a}=\frac{K\lambda }{\beta Cos\theta }$$

where *K* is the Scherrer constant, *λ* is the wavelength, *β* is the full width at half maximum (FWHM) corresponding to the physical broadening of the fibers, and θ is the Bragg’s angle.

Contact angle evaluation of epoxy droplets on single CF monofilaments, generally referred to as the Carroll method and Wagner method, was performed aided by a Carl Zeiss axio Lab A optical microscope. The obtained images were processed with the Image J software. To measure interfacial shear strength (*IFSS*), fiber roving pull-out tests were performed on a universal testing machine (Emic/Instron 23-5D). The samples were prepared by placing the bundle in a perpendicular position in relation to a block of epoxy cured in a silicon mold. Ten samples of each type were tested at a strain rate of 1 mm/min. The load curves were initially linear during the test when the fiber–matrix interface remained intact. The actual pull-out of the fiber bundle from the matrix takes place when the shear forces exceed the critical (peak) load, which is used in Equation (3) to calculate the *IFSS* [34].

(3) $$IFSS=\frac{F_{max}\;}{\;\pi \cdot d_{fb}\cdot l_{e}}$$

where *F_max_* is the maximum pull-out force, and *d_fb_* (2.17–2.93 mm) and *l_e_* (3.15–3.88 mm) are the diameter and length of the fiber bundle embedded in epoxy, which were measured with a digital caliper (accuracy = 0.0125 mm).

## 3. Results and Discussion

### 3.1. Interfacial Shear Strength

**Figure 2:**
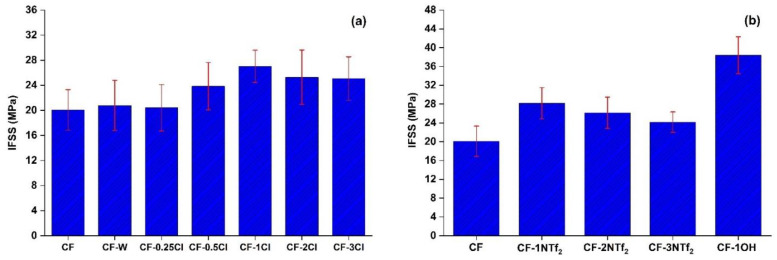
Interfacial shear strength of (**a**) CF, CF-W and CF-Cl (at different concentrations); and (**b**) CF, CF-NTf_2_ (at different concentrations) and CF-1OH.

The pull-out test was used to identify the most promising concentrations of IL since it is an effective and suitable method to evaluate a critical feature of composites, the fiber–matrix interfacial bonding characteristics. Load-displacement pull-out curves are given as supplementary data (Figure S1). The carbon fiber-epoxy matrix IFSS results are compiled in Figure 2, showing the values obtained with CF, CF-W, CF-Cl, CF-NTf_2_, and CF-OH. The pristine CF had an IFSS of 20.08 MPa, which is quite comparable to the value reported in literature for a fiber bundle pullout test of unsized CF and epoxy [35]. Regarding the chosen method for carbon fiber treatment, the effect of ethanol washing (CF versus CF-W) was verified and found to be negligible. Initially, to get an optimized IL content for the surface treatment of CF, various percentages (1, 2 and 3% *w*/*v*) were studied with the ILs C_4_MImCl and C_4_MImNTf_2_. For both ILs, CF treated with 1% *w*/*v* of IL (CF-1Cl and CF-1NTf_2_) produced the most enhanced effect on IFSS. Lower contents of C_4_MImCl (0.25% and 0.50% *w*/*v*) were also tested for the surface modification of CF, but the content of 1% *w*/*v* IL was the optimum and most effective in terms of property improvement. From this outcome, the detailed study was designed to study the effect of 1% *w*/*v* of IL on the surface properties of CF.

Apart from the contact angle, various other factors like increased crosslinking of the epoxy matrix and microcrystalline morphology impact the interfacial properties significantly [36,37]. In this work, that effect can be initially assessed based on the effectiveness of π-bridging between carbon fiber and IL influencing bonding at the interface and also the reactive characteristics of ILs during the curing reaction of epoxy, contributing to its interfacial strength [38]. The interfacial strength is further influenced by the presence of a specific anion and a functional group in the imidazolium *N*-alkyl side chain. The presence of a nucleophilic functional group, OH, enhanced interaction of the fiber with the matrix, most likely due to its ability to react with epoxide groups (Figure 2) [39]. This increased the IFSS value to a maximum of 38.38 MPa with CF-1OH, which is the same effect as the treatment with aqueous ammonia and oxidation of carbon fiber [34,40,41].

The alkyl side chain on the imidazolium cation, on the other hand, has its own significance, as it has low interionic interactions, which allows a more uniform distribution over the CF surface with less agglomeration [38]. The enhanced contribution of side alkyl chains at a low percentage of ionic liquid provided a more balanced approach towards the modification process in terms of imparting plasticity and elastic deformation in order to distribute shear stress gradient along the interface, achieving a toughened composite. A stronger interface bonding would also improve the aging resistance and conduction properties of the rCF composite [42]. This lab scale treatment was chosen considering the possibility of its easy adoption for large scale processes, which can be utilized for the modification of recycled CF that is generally available as random fibers.

### 3.2. Wettability

**Figure 3:**
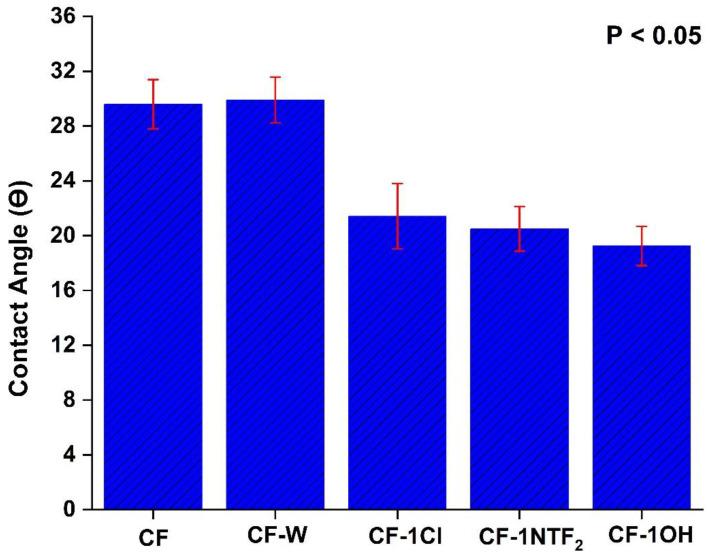
Contact angles of epoxy resin: CF, CF-1Cl, CF-1NTf_2_ and CF-1OH.

To further analyze the effect of IL on wettability and interfacial properties between CF and epoxy, contact angle values were obtained for CF treated with the different ILs at the optimized IL content of 1% *w*/*v*. The values of contact angle are presented in Figure 3, showing the average contact angle between CF and the epoxy of 29.5°, which corresponds to poor wettability due to the inert fiber surface. After its treatment with IL, a 27.6%, 30.7% and 34.9% decrease was observed for CF-1Cl, CF-1NTF_2_ and CF-1OH, respectively, indicating enhanced compatibility [43]. This also indicates that the chemical nature of the fiber surface was modified, although only 1% *w*/*v* of IL was used. The maximum decrease in contact angle shown by CF-1OH could be due to the presence of a functional group with polar characteristics that might enhance its surface polarity compared to the other samples, enabling dipole-dipole interactions and hydrogen bonding. Indeed, better compatibility between epoxy and CF from the lower contact angle due to high surface activity and functionality favors chemical and physical interlocking, contributing to the interfacial adhesion in the composite [44], helping to justify the previous IFSS results.

**Figure 4:**
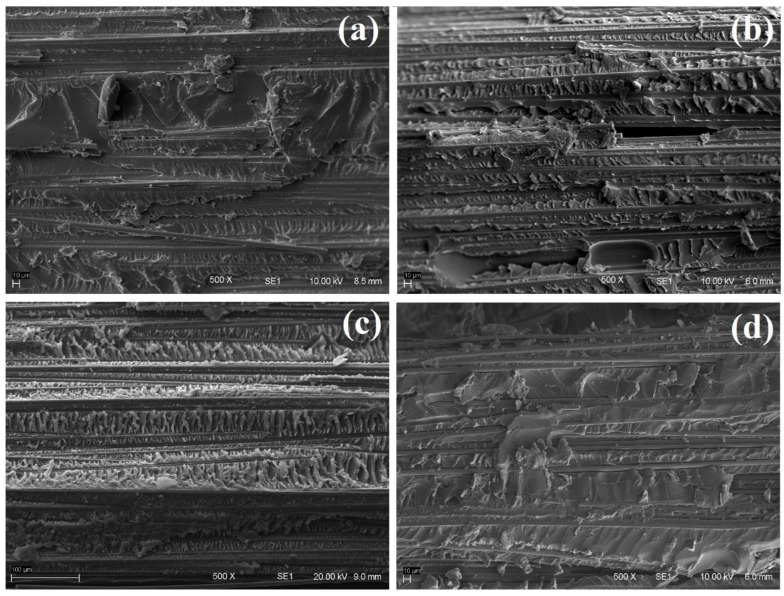
SEM micrographs of the interfacial pull-out region of the samples: (**a**) CF, (**b**) CF-1Cl, (**c**) CF-1NTf_2_ and (**d**) CF-1OH.

The morphological analysis of the interfacial pull-out region of the samples carried-out to assess epoxy retention and its interaction with the fiber surface is shown in Figure 4. Large break gaps and debonded interfaces due to the shear forces can be seen on CF. The micrograph of CF, without sizing, shows large gaps that could result from relatively weak interface bonding strength and stress transfer efficiency. Clearly, a dense matrix layer was still there after the pull-out for CF-1Cl, CF-1NTf_2_ and CF-1OH, which resulted from a relatively stronger interaction with the fiber. The serrated bonding aspects were also evident at the surface of IL-modified samples, suggesting increased energy absorption leading to composite toughening [34].

### 3.3. Surface Analysis

**Figure 5:**
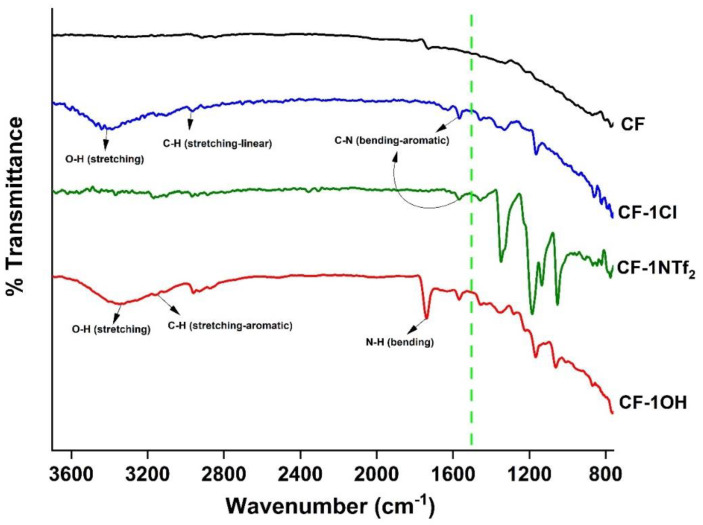
FTIR-ATR transmittance spectra for the studied CF samples.

After the modification of CF with IL, a weight gain was observed, being 10.94%, 9.34% and 7.38% for CF-1Cl, CF-1NTf_2_ and CF-1OH, respectively. In addition, among all the studied concentrations, the increase in weight was maximum for CF-Cl with only 1% *w*/*v* of IL, suggesting an optimized amount of IL on the fiber surface that triggered a change in surface properties. Figure 5 shows the FTIR-ATR transmittance spectra of CF, CF-1Cl, CF-1NTf_2_ and CF-1OH, and the transmittance peaks of the studied ILs are detailed as supplementary material (Tables S1–S3) to aid in the discussion. The CF spectrum shows a straight line with no transmittance peak due to the absence of any functional group on the CF surface. After the surface treatments with 1% *w*/*v* of IL, various transmittance peaks associated with IL were observed. In the spectrum of CF-1NTf_2_, peaks due to aromatic stretching of the imidazolium ring can be seen at 3121 cm^−1^ and 3158 cm^−1^. Specific peaks related to NTf_2_ appeared at 1349 cm^−1^ (O–S–O, stretching) and 1056 cm^−1^ (S-N-S, stretching), and there are other peaks at 2878 cm^−1^, 2941 cm^−1^ and 2968 cm^−1^ that belong to C-H (stretching) of the linear alkyl chain attached to the imidazolium ring. The peak present at 1571 cm^−1^ is due to the bending of C-N in the aromatic ring [45,46,47].

For CF-1Cl, in addition to the peaks (2973 cm^−1^, 2870 cm^−1^, 1600 cm^−1^) corresponding to the imidazolium ring and linear carbon chain, a broad peak at around 3200 cm^−1^ is present, which corresponds to the OH stretching. A broad peak for OH reveals hydrogen bonding corresponding to the water molecule [5]. For C_2_OHMImCl, the peak at 1340 cm^−1^ is due to the bending vibration of the OH functional group, whereas the peak at 3316 cm^−1^ is due to OH stretching, indicating its hydrophilic nature. The attachment of IL to the CF surface suggests a favorable interaction between them, which may arise from the delocalized electronic cloud of the IL’s imidazolium ring and sp^2^ hybridized carbon atoms in the hexagonal structure of CF [48].

**Figure 6:**
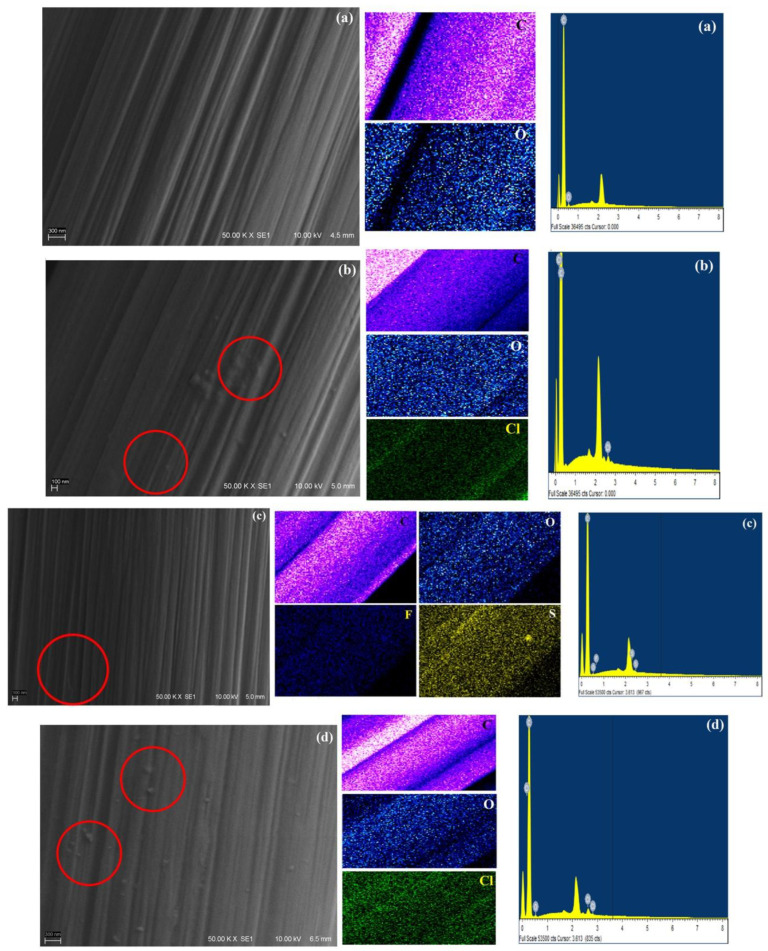
SEM micrographs, elemental maps and EDS spectrum of the samples: (**a**) CF, (**b**) CF-1Cl, (**c**) CF-1NTf_2_ and (**d**) CF-1OH.

The surface morphology of CF samples analyzed with SEM micrographs is shown in Figure 6. The facile way adopted to modify the CF with ILs produced a significant change on its surface. The micrographs show a smooth surface containing carbon fiber having some inherently available oxygen as shown in EDS. The presence of IL can be observed as a dispersed phase of nanostructured particles attached and uniformly distributed over the surface of the CF without affecting its surface topography in CF-1Cl, CF-1NTf_2_ and CF-1OH. Careful observation shows that the morphology of the surface of IL-treated CF was different. C_4_MImCl was present on the surface as small irregular-shaped particles and even as some aggregates. C_4_MImNTf_2_ was present on the surface as a layered type of structure, whereas C_2_OHMImCl appeared as a circular particle, comparatively larger than the others.

Elemental analysis of the carbon fiber surfaces showed the presence of specific elements belonging to the ILs used. The compositional maps showed chlorine for CF-1Cl, sulfur and fluorine for CF-1NTf_2_, and chlorine for CF-1OH, in addition to carbon and oxygen, being distinguishable characteristics to affirm the presence of the corresponding ILs on the CF surface. The attachment of IL to CF may be possible due to π-π bond stacking, and this is the only apparent mechanism that can provide interaction [30]. Flexibly, present on the surface of carbon fiber, its molecular component will provide a bonding mechanism with the epoxy matrix.

An X-ray diffraction analysis was carried out to investigate the effect of IL on the crystallite size and microstrains of CF. The crystallite size is basically the mean size of coherent-scattering regions in a specified direction of CF. The XRD spectra of the fibers show two discernible peaks, one at 2*θ* = 43.9°, corresponding to the 002 plane that is perpendicular to the graphite layers (perpendicular to the fiber axis), and one at 2*θ* = 24.8°, representing the 100 planes along the graphite layers. The crystallites are mainly oriented along the fiber axis and the 002 plane basically reflects its turbostratic structure.

**Figure 7:**
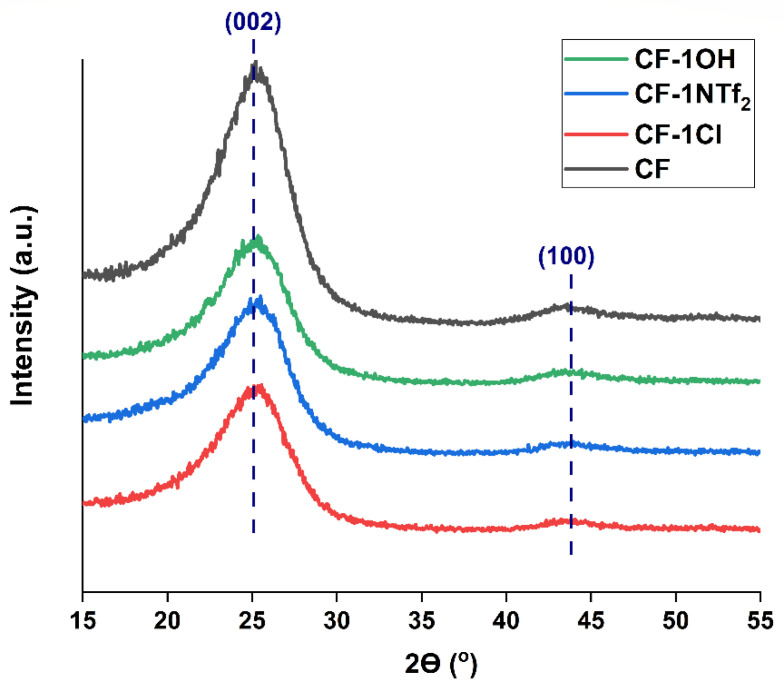
XRD spectrum of the studied samples and zoomed-in view of the 002 peak.

The average crystallite size (L_c_) in the direction of the 002 plane is within 2.07–1.89 nm and the crystallite size (L_a_) along the 100 plane is within 1.87–1.94 nm. Both the crystallite size and microstrain have been calculated from FWHM and by using Scherrer’s equation. A broad peak belonging to the 002 plane is observed for CF, whereas the broadening is reduced for CF-1Cl, CF-1NTf_2_ and CF-1OH, as shown in Figure 7. The crystallite size calculated for CF along L_c_ and L_a_ is 2.04 nm and 1.87 nm, respectively. The crystallite size (L_c_) was reduced for CF-1Cl, CF-1NTf_2_ and CF-1OH, whereas a slight increase in L_a_ can be seen compared to CF. This improved microstructure behavior of CF due to IL means increased surface area of the graphitic structure along basal planes. The alignment of the hexagonal graphitic structure along the basal plane has the potential to enhance the electrical and mechanical properties at the fiber surface. Better ordering of the graphitic microstructure in both directions has contributed to the decrease in strain values [43,49]. An increased crystallite size (L_a_) decreased the strain between the layers of carbon atoms and both of these effects have resulted in a broadening of the peak depicting a more relaxed microstructure [50].

### 3.4. Electrical Properties

Carbon fiber-based composites are suited for multifunctional applications due to their inherent electrical and mechanical properties, and it was a primary objective of this study to increase CF electrical conductivity. The obtained values of electrical resistivity and contact resistance are shown in Table 1, being 11.30 μAm and 325 Ω for the carbon fiber, with 29% lower than the manufacturer’s value (SIGRAFIL C T50-4.8/280-UN), which is acceptable for comparison purposes [51].

After IL treatment, the CF electrical resistivity was reduced, which can be explained by analyzing the constituents (cation, anion, alkyl side chain, functional group) of the ILs, being attributed to a more ordered structure long-range ion pair distribution on the fiber surface [52]. It is worthwhile to discuss the different characteristics of the hydroxyl functional group (OH) on the alkyl side chain, where the presence of polar groups enhances the surface polarizability in the presence of an electrical field due to an uneven distribution of charges. The polarity of the functional group (OH greater than CH_3_) influences the overall electrical transport mechanism of the IL [51].

The contribution of ILs towards conductivity can be explained by considering the electron conduction dominancy over the ion contribution in the dry state. Primarily, the use of imidazolium cation showed its good charge carrying ability, which is more effective in the parallel direction of the imidazolium ring than in the perpendicular direction. The reduced electrical resistivity of CF-1NTF_2_ compared to CF-1Cl is due to its presence as a layered structure on a planar surface, influencing the differential capacitance inside the layers [53]. In the case of NTf_2_, the anion has a greater ability to diffuse and form multiple H-bonds via oxygen atoms in addition to having a strong delocalized negative charge, which reduces the hydrogen bonding with cation and contributes to charge carrying for electrical conduction [54]. The low percentage of IL (1% *w*/*v*) in the present case, provides reduced ion pairing or aggregation, which increases the number of available charge carriers and its mobility. Compared to CF-1Cl and CF-1OH, the reduced electrical resistivity of CF-1OH is due to better packing with smaller anion and polar nature from the presence of the OH group [2,55].

**Figure 8:**
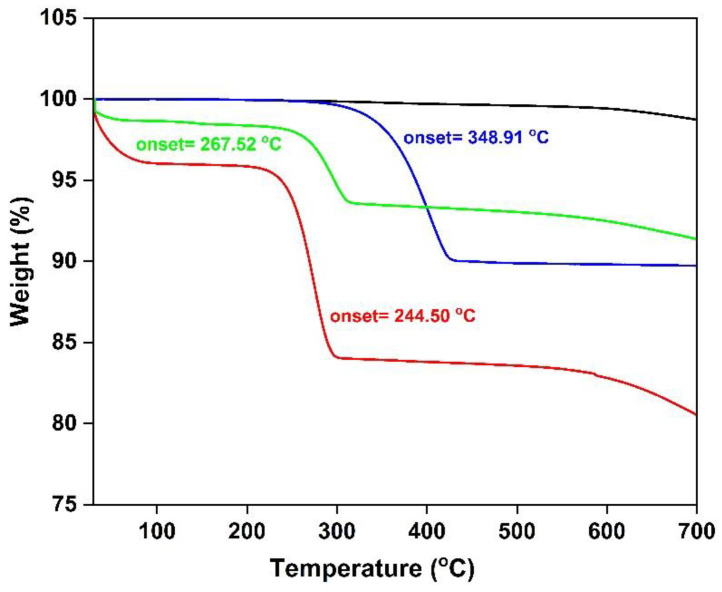
TG results for CF (black), CF-1Cl (red), CF-1NTf_2_ (blue) and CF-1OH (green).

The thermal stability of CF and IL-modified CF has been determined by thermogravimetric analysis (Figure 8). CF is thermally stable up to 600 °C, and the thermal stability of IL-modified CF is mainly dependent on the thermal stability of the particular IL [56]. Initially, loss of water has been observed in CF treated with chloride ILs. The onset temperature of degradation of CF-1NTF_2_ is higher compared to that of CF-1Cl and CF-1OH, which can be attributed to the anion. Overall, the IL-treatment provided CF with thermal stabilities that are high enough for common CF/epoxy composite applications.

## 4. Conclusions

A procedure for the surface treatment of CF has been developed, in which an optimized concentration of 1% *w*/*v* IL was identified. A large increase in the IFSS between CF and epoxy was obtained with the IL 1-(2-hydroxyethyl)-3-methylimidazolium chloride, showing potential use for advanced composites. Enhanced electrical conductivity on the fiber surface was also obtained, allowing the design of composites with more electrically conductive interfaces. The obtained characteristics provide an opportunity to use carbon fiber, including recycled carbon fiber, in wider multifunctional applications.

## Figures and Tables

**Table 1:** Electrical resistivity and contact resistance of the CF samples.

	Resistivity (μAm)	Contact Resistance (Ω)
CF	11.30 ± 0.14	325
CF-1Cl	8.39 ± 3.68	244
CF-1NTF_2_	3.33 ± 0.64	297
CF-1OH	2.84 ± 0.28	284

## Data Availability

Data is available in the article and Supplementary Materials.

## Supplementary Materials

The following supporting information can be downloaded at: https://www.mdpi.com/article/10.3390/molecules27207001/s1, Figure S1: Load-displacement pull-out curves of CF, CF-1Cl, CF-1NTf_2_ and CF-1OH; Figure S2: Curve fitting of two terminal electrical resistance vs. distance for CF, CF-1Cl, CF-1NTf_2_ and CF-1OH; Table S1: FTIR transmittance peaks of C4MImCl [5,45,46,47]; Table S2: FTIR transmittance peaks of C_4_MImNTf_2_ [45,46,47]; Table S3: FTIR transmittance peaks of C_2_OHMImCl [5,45,46,47].
