# Supplementary material for: Multifunctional Characteristics of Carbon Fibers Modified with Imidazolium Ionic Liquids
Source: Molecules. 2022 Oct 18;27(20):7001. doi: 10.3390/molecules27207001 (PMC9611141; doi:10.3390/molecules27207001)
Supplement: Supplementary file 1 [file molecules-27-07001-s001.zip › molecules-1920014-supplementary.pdf]

MULTIFUNCTIONAL CHARACTERISTICS OF CARBON FIBERS MODIFIED WITH IMIDAZOLIUM IONIC LIQUIDS

Bilal Ghafoor[1], Henri Stephan Schrekker[2], Sandro Campos Amico[1,*]

[1]PPGE3M, Federal University of Rio Grande do Sul, Porto Alegre/RS, 91501-970, Brazil

[2]Laboratory of Technological Processes and Catalysis, Institute of Chemistry, Federal University of Rio Grande do Sul, Porto Alegre/RS, 91501-970, Brazil

*Correspondence: amico@ufrgs.br

SUPPLEMENTARY MATERIAL

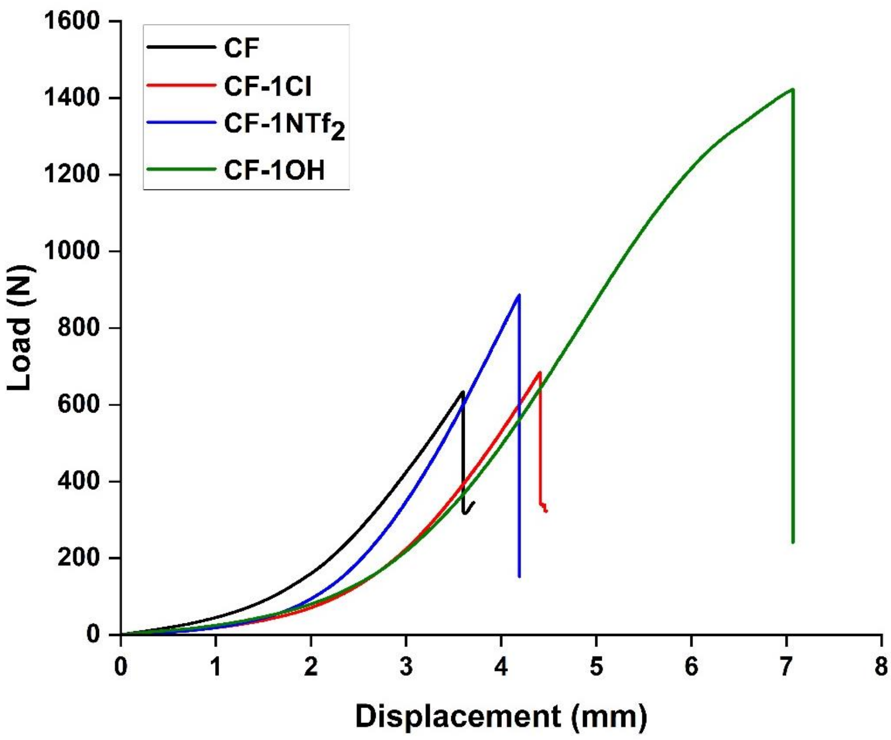

Figure S1. Load-displacement pull-out curves of CF, CF-1Cl, CF-1NTf$_2$ and CF-1OH.

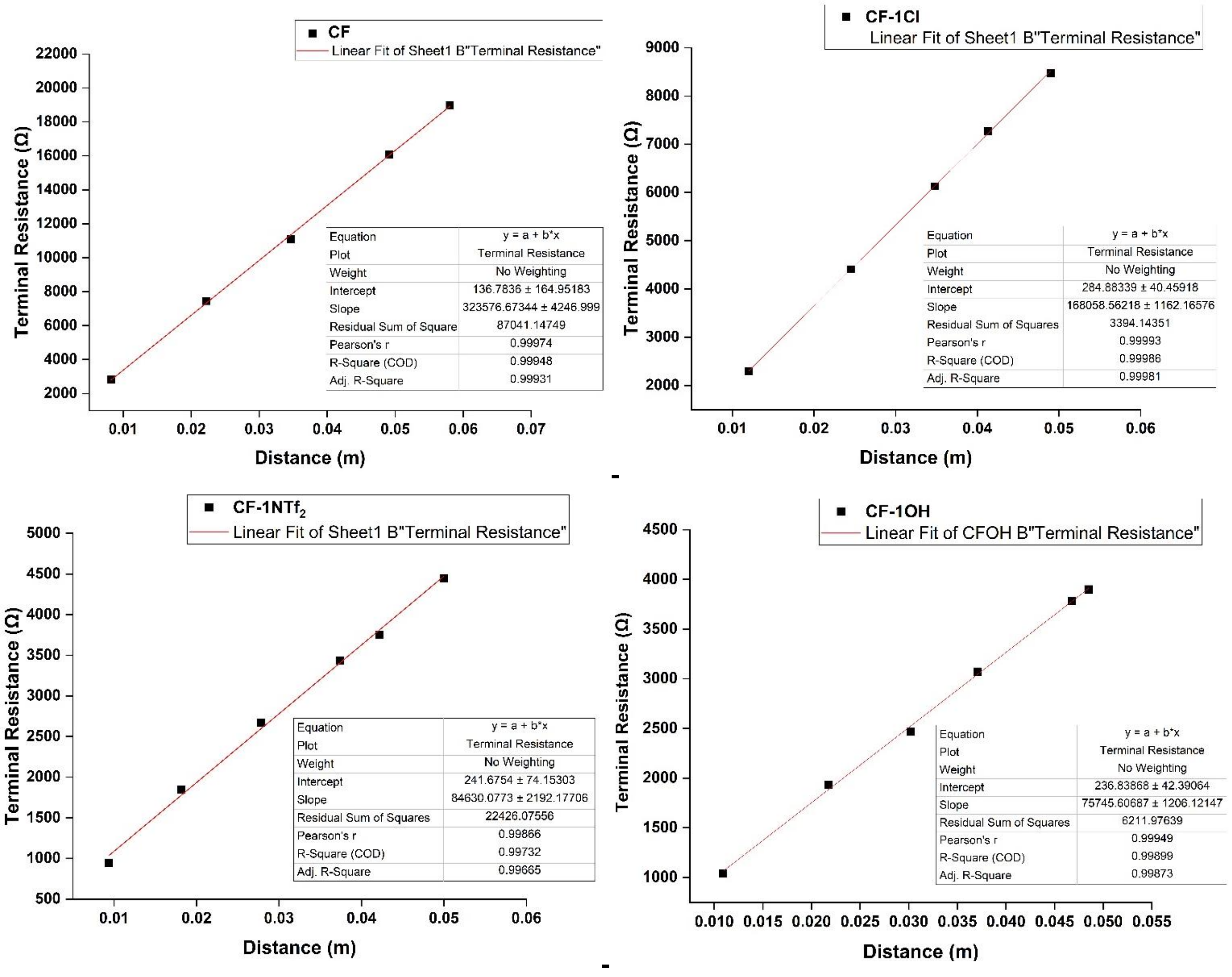

Figure S2. Curve fitting of two terminal electrical resistance vs distance for CF, CF-1Cl, CF-1NTf$_2$ and CF-1OH.

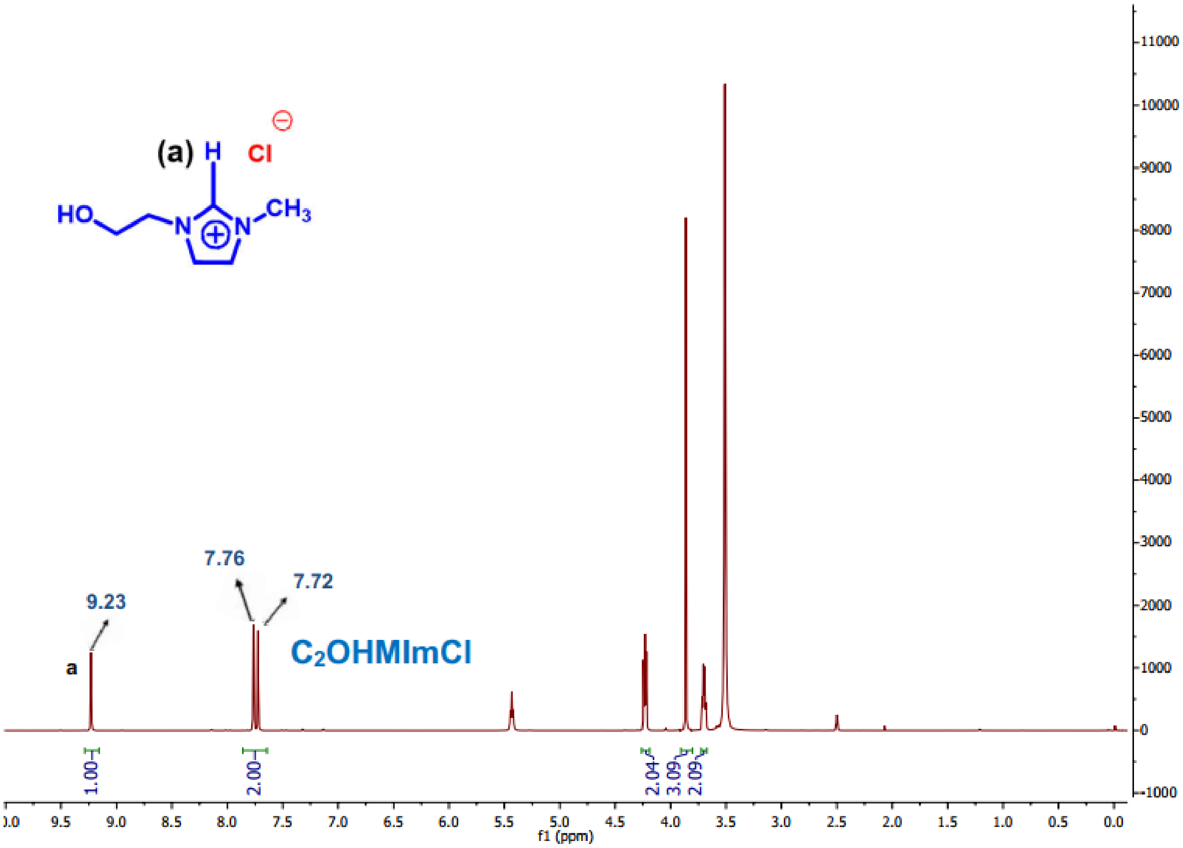

Figure S3. ^{1}H NMR spectrum of 1-(2-hydroxyethyl)-3-methylimidazolium chloride, $C_2OHMImCl$.

Table S1. FTIR transmittance peaks of C_4MImCl [5, 45-47].

**Wavenumber of the bands**	
**Wave number (cm^{-1})**	**Description**
3300	O-H (stretching)
2973, 2870	C-H (stretching-linear)
1635	C=C (stretching)
1600	N-H (bending)
1573	C–N (bending-aromatic)
1169	C-H (stretching-aromatic)
1166	C–H (bending-aromatic)
840	C-H (bending)

Table S2. FTIR transmittance peaks of $C_4MImNTf_2$ [45-47].

**Wave number (cm^{-1})**	**Description**
3121, 3158	C-H (stretching-aromatic)
2878, 2941, 2968	C-H (stretching-linear)
1571	C–N (bending-aromatic)
1466, 1437	C-C (stretch-aromatic), C-H (scissoring)
1349	O–S–O (stretching)
1186, 1138	C-N (stretching), C–H (scissoring)
1056	S-N-S (stretching)
790	C–H, -HC=CH- (bending)
883	C-H (bending)

Table S3. FTIR transmittance peaks of $C_2OHMImCl$ [5, 45-47]

**Wave number (cm^{-1})**	**Description**
3316	O-H stretching vibration
3148, 3097	C-H (stretching-aromatic)
2959, 2874	C-H (stretching-linear)
1568	C–N (bending-aromatic)
1450	C-C (stretch-aromatic)
1340	O-H (bending)
1258	C-N stretching (aromatic)
1167, 1069	C-H (bending), C-N (stretching)
868	C-H (bending)
